# Glycopeptidolipid of *Mycobacterium smegmatis* J15cs Affects Morphology and Survival in Host Cells

**DOI:** 10.1371/journal.pone.0126813

**Published:** 2015-05-13

**Authors:** Nagatoshi Fujiwara, Naoya Ohara, Midori Ogawa, Shinji Maeda, Takashi Naka, Hatsumi Taniguchi, Saburo Yamamoto, Minoru Ayata

**Affiliations:** 1 Department of Food and Nutrition, Faculty of Contemporary Human Life Science, Tezukayama University, Nara City, Nara, Japan; 2 Department of Bacteriology, Osaka City University Graduate School of Medicine, Osaka City, Osaka, Japan; 3 Department of Oral Microbiology, Okayama University Graduate School of Medicine, Dentistry and Pharmaceutical Sciences, Okayama City, Okayama, Japan; 4 Department of Microbiology, School of Medicine, University of Occupational and Environmental Health, Kitakyushu City, Fukuoka, Japan; 5 Molecular Epidemiology Division, Mycobacterium Reference Center, The Research Institute of Tuberculosis, Japan Anti-Tuberculosis Association, Kiyose City, Tokyo, Japan; 6 MBR Co. Ltd., Toyonaka City, Osaka, Japan; 7 Japan BCG Laboratory, Kiyose City, Tokyo, Japan; 8 Department of Virology, Osaka City University Graduate School of Medicine, Osaka City, Osaka, Japan; Centre National de la Recherche Scientifique—Université de Toulouse, FRANCE

## Abstract

*Mycobacterium smegmatis* has been widely used as a mycobacterial infection model. Unlike the *M*. *smegmatis* mc^2^155 strain, *M*. *smegmatis* J15cs strain has the advantage of surviving for one week in murine macrophages. In our previous report, we clarified that the J15cs strain has deleted apolar glycopeptidolipids (GPLs) in the cell wall, which may affect its morphology and survival in host cells. In this study, the gene causing the GPL deletion in the J15cs strain was identified. The *mps1-2* gene (MSMEG_0400-0402) correlated with GPL biosynthesis. The J15cs strain had 18 bps deleted in the *mps1* gene compared to that of the mc^2^155 strain. The *mps1*-complemented J15cs mutant restored the expression of GPLs. Although the J15cs strain produces a rough and dry colony, the colony morphology of this *mps1*-complement was smooth like the mc^2^155 strain. The length in the *mps1*-complemented J15cs mutant was shortened by the expression of GPLs. In addition, the GPL-restored J15cs mutant did not survive as long as the parent J15cs strain in the murine macrophage cell line J774.1 cells. The results are direct evidence that the deletion of GPLs in the J15cs strain affects bacterial size, morphology, and survival in host cells.

## Introduction

Tuberculosis is an infectious disease worldwide, and the causative agent, *Mycobacterium tuberculosis*, is a slow-growing intracellular pathogen. To control mycobacterial infection, it is necessary to clarify the mechanisms of host–pathogen interactions and how the pathogen survives in host cells.

*Mycobacterium smegmatis* is a rapidly growing nonpathogenic mycobacterium. Its features make it useful as a tool for genetic analysis in the research on mycobacterial infection. In particular, the *M*. *smegmatis* mc^2^155 strain is widely used because of its high rate of transformation. We screened another *M*. *smegmatis* strain, J15cs, and constructed a convenient and safe host-vector system of a mycobacterial model. We previously reported that the J15cs strain replicated our original vector, pYT923, and survived in host cells longer than the mc^2^155 strain [1]. We clarified a further difference between J15cs and mc^2^155 strains. The J15cs strain lacks glycopeptidolipids (GPLs) on the cell surface, and this may affect its morphology and survival in host cells [2].

GPL, also called C-mycoside, is produced in the outer layer of some non-tuberculous mycobacteria, including *Mycobacterium avium*, *M*. *intracellulare*, *M*. *scrofulaceum*, *M*. *abscessus*, *M*. *chelonae*, and *M*. *smegmatis*. Structurally, GPLs comprise a lipotetrapeptide core, fatty acid, and oligosaccharide. Three amino acids (D-phenylalanine, D-*allo*-threonine and D-alanine) link a 3-hydroxy or 3-methoxy long-chain fatty acid (C26-34) to the *N*-terminal and an amino alcohol, alaninol, to the *C*-terminal. The 6-deoxy-talose is glycosidically linked to the D-*allo*-threonine and a methylated L-rhamnose to the alaninol to generate the non-specific core GPLs found in all members of GPL-producing mycobacteria. In *M*. *avium* and *M*. *intracellulare*, an oligosaccharide unit is elongated from 6-deoxy-talose, leading to the serotype-specific, so-called polar GPLs [3]. It is considered that GPLs are related to bacterial aggregation, sliding motility, biofilm formation, and cell wall integrity [4,5].

In this study, we examined the GPL biosynthesis pathway and clarified the causative gene cluster that deletes the GPLs in the J15cs strain. We targeted the *mps* gene that involves the tetrapeptide synthesis in the GPLs [6]. Interestingly, recovery of the GPLs affected colony morphology and survival in host cells.

## Materials and Methods

### Bacterial strains

The J15cs strain is diverged from the *M*. *avium* Jucho strain and maintained in our laboratory [1,2]. The mc^2^155 strain (ATCC 607) was purchased from the American Type Culture Collection (Manassas, VA). Both strains were grown on Middlebrook 7H11 agar (Difco Laboratories, Detroit, MI) with 0.5% glycerol and 10% Middlebrook OADC enrichment (Difco Laboratories) or nutrient agar at 37°C for 2–7 days. *Escherichia coli* was cultured in LB broth. Kanamycin (20 μg/ml) was added to the selective medium for pNN2-containing plasmids.

### Amplification and sequencing of DNA fragments in *M*. *smegmatis* J15cs genome

DNA fragments containing the region covering the *mps1* and *mps2* genes were amplified by polymerase chain reaction (PCR) with specific primer pairs. The primers were prepared in reference to the reported sequence of the mc^2^155 strain (Table 1). KOD FX Neo DNA polymerase (Toyobo Co., Ltd, Osaka, Japan) was used for PCR amplification and the condition for PCR was 95°C-2 min, 5 cycles of 98°C-10 sec, 72°C-2.5 min, 5 cycles of 98°C-10 sec, 70°C-2.5 min, 30 cycles of 98°C-10 sec, and 68°C-2.5 min. The expected sizes of PCR products were 4,240 bps (primer pair: MSMEG_0400-F1 and MSMEG_0400-R2), 3,758 bps (primer pair: MSMEG_0400-F2 and MSMEG_0400-R1), 2,987 bps (primer pair: MSMEG_0401-F1 and MSMEG_0401-R1), 4,204 bps (primer pair: MSMEG_0402-F1 and MSMEG_0402-R2), and 3,776 bps (primer pair: MSMEG_0402-F2 and MSMEG_0402-R1). The DNA fragments were isolated and purified with a gel extraction kit (Qiagen, Germantown, MD) and were directly sequenced in both directions with series of synthetic primers (not shown), a sequencing kit (BigDye terminator v3.1 cycle sequencing kit, Life Technologies, Carlsbad, CA), and automated sequencers (Prism 310 or 3130xl genetic analyzer, Life Technologies).

**Table 1:** Sequence of primers used for amplifying MSMEG_0400–0402 in this study.

Primer	Sequence (5´ to 3´)
MSMEG_0400-F1	ATGTCCGCTGAACAGCCGGGAGAAAC
MSMEG_0400-F2	ACGCGTCAACTGCTCGGTGAAGTACC
MSMEG_0401-F1	AAACGCCGTGGAGGAACTGCTCACCG
MSMEG_0402-F1	CGAACGTCTTCGATCGGCAACGCATC
MSMEG_0402-F2	CACAGCACACCACCATCAACACCGTG
MSMEG_0400-R1	ACCACCTGCATCGAGGAGATGCTGTC
MSMEG_0400-R2	GGTACTTGGTGGTGAACCAACCGACG
MSMEG_0401-R1	ATGCCGTGTCGTGGCGAATC
MSMEG_0402-R1	ACGAACAGTGCCAACCCGAGTATGTC
MSMEG_0402-R2	GGTGTTGATCAACAGGCCCACCATCG
Nde-mps1	GGCATATGGAACCTGTCGACGGGGCTCTACCGCTGTC
Pac-mps1	GGCCTTAATTAATCAGATGACCCCGAGGCCCACGATCAACTCCCAG

### Construction of plasmids expressing *mps1* and transduction to *M*. *smegmatis* J15cs strain

DNA fragments containing the whole *mps1* gene of the mc^2^155 strain were amplified by PCR with specific primer pairs (Primer Nde-mps1 and Pac-mps1) in Table 1. LA Taq DNA polymerase (Takara Bio Inc., Shiga, Japan) was used for PCR amplification. The PCR conditions were 94°C for 1 min, followed by 98°C for 10 sec, 68°C for 15 min, which lasted for 30 cycles, and a final extension at 72°C for 10 min. The DNA fragments were purified and cloned into a pGEM-T Easy vector (Promega Corp., Fitchburg WI). A plasmid clone containing the authentic sequence was obtained (a single mutation was allowed because it is silent). The 10.3 kb of amplified DNA were double digested with *NdeI*-*PacI* and ligated into the same sites of pNPP, resulting pNPP-MPS1. The pNPP is a pUC19 derivative containing the promoter region of *aphII* and the terminator of the antigen gene from *Mycobacterium kansasii* [7]. Then, pNPP-MPS1 was digested with *XbaI* and the inserted fragment was ligated into the same site of an *E*. *coli*-mycobacteria shuttle plasmid pNN2 to yield pNN-MPS1 [8]. The constructed plasmid, pNN-MPS1, was selected and its inserts were confirmed.

### Analysis of GPL compositions

The GPLs were detected by thin-layer chromatography (TLC) as described previously [2]. Briefly, the heat-killed bacteria were sonicated, and crude lipids were extracted with chloroform-methanol (2:1, vol/vol). The crude lipids were hydrolyzed with 0.2 N sodium hydroxide in methanol at 37°C for 2 h, and neutralized with 6 N hydrochloric acid. Alkaline-stable lipids extracted in the organic phase of a two-layer partition with chloroform-methanol (2:1, vol/vol) and water, were evaporated and precipitated with acetone to remove any acetone-insoluble components. The TLC on Silicagel G (Uniplate; 20 by 20 cm, 250 μm; Analtech, Inc., Newark, DE) was developed with a solvent system of chloroform-methanol (90:10, vol/vol). The TLC plate was sprayed with 20% sulfuric acid in ethanol and charred at 180°C for 3 min. The GPLs were detected as a brownish-yellow spot. The GPLs were purified by exposing the TCL plate to iodine vapor, and the GPL spot was marked, scraped off, and eluted with chloroform-methanol (2:1 vol/vol). To assign the structures of the GPLs, the molecular weight of each GPL was measured by matrix-assisted laser desorption/ionization time-of-flight mass spectrometry (MALDI-TOF MS) using an Ultraflex II (Bruker Daltonics, Billerica, MA) with 10 mg/ml 2,5-dihydroxybenzoic acid (DHB) in chloroform-methanol (1:1, vol/vol) as a matrix, and analyzed in Reflectron mode with an accelerating voltage operating in positive mode at 20 kV.

### Colony morphology and scanning electron microscopy

Colony morphology was observed using an optical microscope after incubation for 10 days on nutrient agar plates. To prepare the bacteria for scanning electron microscopy (SEM), they were suspended with 0.1 M phosphate buffer (pH 7.2), passed through a 5 μm membrane filter (Millipore, Billerica, MA, USA) to remove aggregates, and then put on coverslips (Thermanox Plastic, 13 mm diameter; Thermo Fisher Scientific, Rochester, NY) coated with 0.1% (w/v) poly-L-lysine solution. The bacteria were fixed with 2.5% glutaraldehyde (TAAB Laboratories Equipment Ltd., West Berkshire, UK) in 0.1 M phosphate buffer for 2 h at room temperature, washed twice with 0.1 M phosphate buffer, and post-fixed with 1% osmium tetroxide (TAAB Laboratories Equipment) in 0.1 M phosphate buffer for 1 h at room temperature. The bacteria were then dehydrated in a graded ethanol series. After permutation with 3-methyl butyl acetate, the critical point drying was determined using a Hitachi HCP-2 Critical Point Dryer (Hitachi High-Technologies Corp., Tokyo, Japan). SEM was performed at ×5,000 on a Hitachi FE-SEM S-4700 instrument (Hitachi High-Technologies Corp.) using an accelerating voltage of 10 kV [9].

### Intracellular growth in J774.1 cells

A murine macrophage, J774.1 cell line (Cell Resource Center for Biomedical Research Institute of Development, Aging and Cancer Tohoku University, Sendai, Miyagi, Japan), was cultured in RPMI 1640 medium (Nissui Pharmaceutical, Tokyo, Japan) supplemented with 10% inactivated fetal calf serum (FCS, GIBCO, Grand Island, NY) at 37°C in 5% CO_2_. The J774.1 cells were grown to semi-confluence for 48 h on 24-well flat-bottom tissue culture plates (Becton Dickinson Labware, Oxnard, CA). *M*. *smegmatis* strains were pre-cultured on nutrient agar plates for 7 days. PBS added on agar plate. Bacterial cells were suspended, and were mixed slowly. After collecting and pumping by 23G syringe, the bacterial suspension was passed through a 5 *μ*m membrane filter to remove aggregates. A single-cell suspension was checked by acid-fast staining, and was prepared at a concentration of approximately 1 × 10^8^ CFU/ml. A bacterial single-cell suspension of 1×10^7^ CFU/well was inoculated into the J774.1 cells at an approximate multiplicity of infection 10. After cocultivation for 3 h, the infected J774.1 cells were washed twice with PBS in order to remove non-infecting bacteria. To kill any remaining extracellular bacteria, the infected J774.1 cells were further incubated for 2 h with fresh culture medium supplemented with 5% inactivated FCS and 200 μg/ml of gentamicin (Sigma Chemicals, St. Louis, MO). The medium was replaced with fresh medium containing 2 μg/ml of gentamicin (this time point was day 0), and the infected cells were cultured for 8 days. The survival of mycobacteria was evaluated by counting colony-forming units (CFU). The adherent cells were treated with 1% Triton-X100/PBS, and were sonicated. The lysates plated on nutrient agar. The experiments were performed in triplicate, and statistical analysis was carried out using Dunnett’s test [1].

## Results

### Mutation of *mps1-2* genes in *M*. *smegmatis* J15cs strain

**Fig 1:**
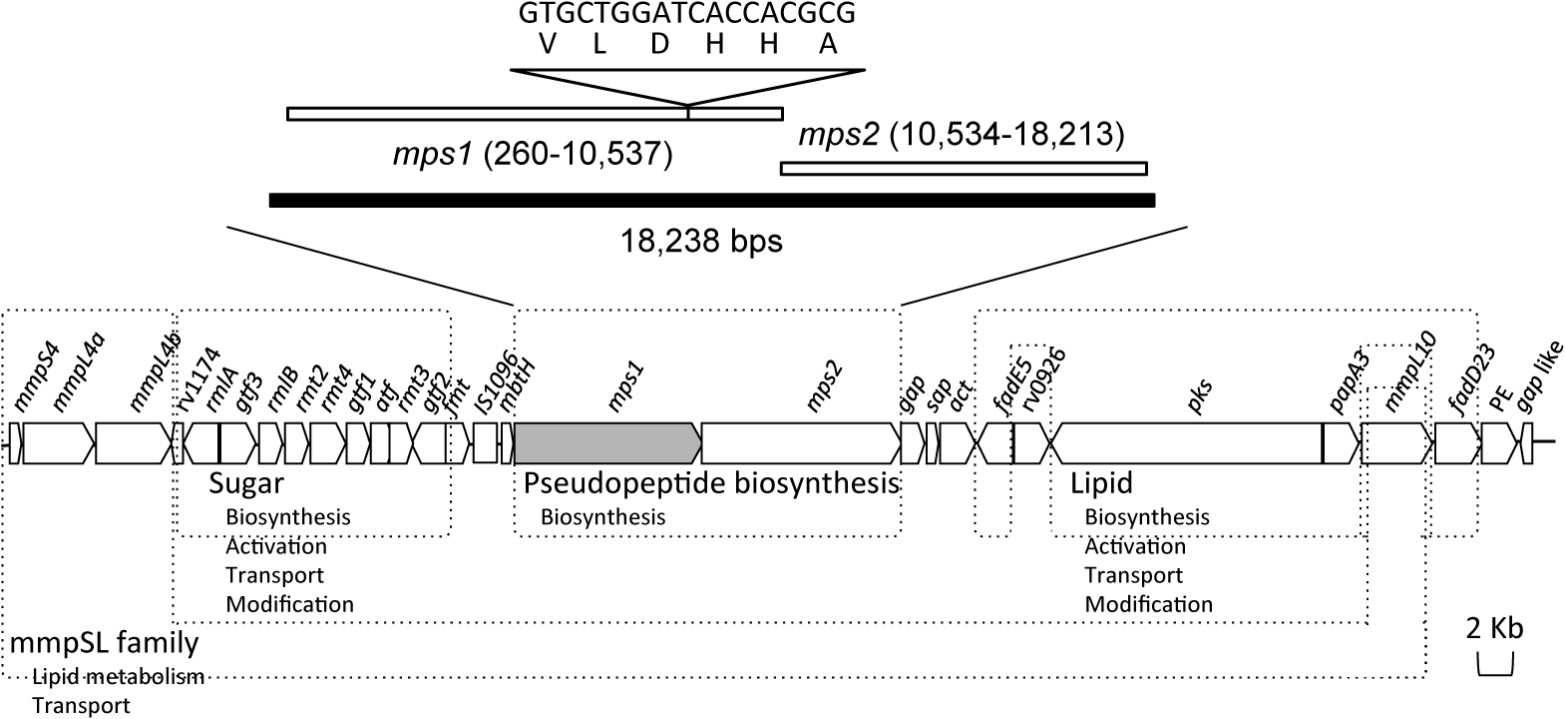
The *mps1* gene of *M*. *smegmatis* J15cs strain was 18 bps shorter than that of *M*. *smegmatis* mc^2^155 strain. The schematic gene map of the GPL biosynthetic cluster in *M*. *smegmatis* [6,13]. The sequence of MSMEG_0400–0402 was determined (GenBank accession no. AB924538). Two open reading frames, *mps1* and *mps2*, were encoded in this segment. The Mps1 of the J15cs strain was 6 consecutive amino acids shorter compared to that of the mc^**2**^155 strain, whereas the Mps2 of the J15cs strain was identical to that of the mc^**2**^155 strain.

The DNA fragments of MSMEG_0400–0402 were amplified by PCR and the sequence was determined. The nucleotide sequence MSMEG_0400–0402 of the J15cs strain was deposited in the NCBI GenBank database under accession number AB924538. The results showed a segment of DNA with a total length of 18,238 bps. The sequence was identical to that reported for the mc^2^155 strain (GenBank accession no. AY439015) [10] except for a gap of 18 consecutive bps nucleotides (Fig 1). Two open reading frames were encoded in this segment, and they corresponded to the protein Mps1 and Mps2. The Mps1 of the J15cs strain implied a 6 consecutive amino acid deletion compared to that of the mc^2^155 strain, whereas the *mps2* sequence of the J15cs strain was identical to that of the mc^2^155 strain.

### Phenotype of *M*. *smegmatis* J15cs mutant complemented with mc^2^155-*mps1*

**Fig 2:**
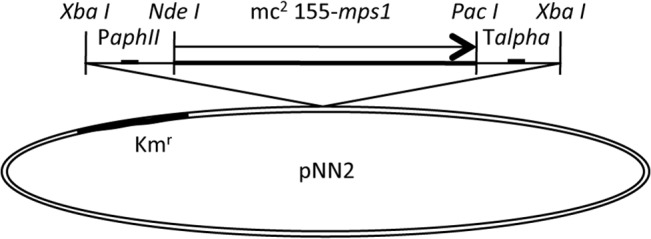
Construction of the mc^2^155-*mps1* plasmid. The *mps1* of the mc^**2**^155 strain was inserted into a pNN2 vector (kanamycin resistance). P*aphII*, the promoter of *aphII*; mc^**2**^155-*mps1*, the open reading frame of *mps1* from *M*. *smegmatis* mc^**2**^155; T*alpha*, the terminator region of the alpha antigen gene from *Mycobacterium kansasii*.

**Fig 3:**
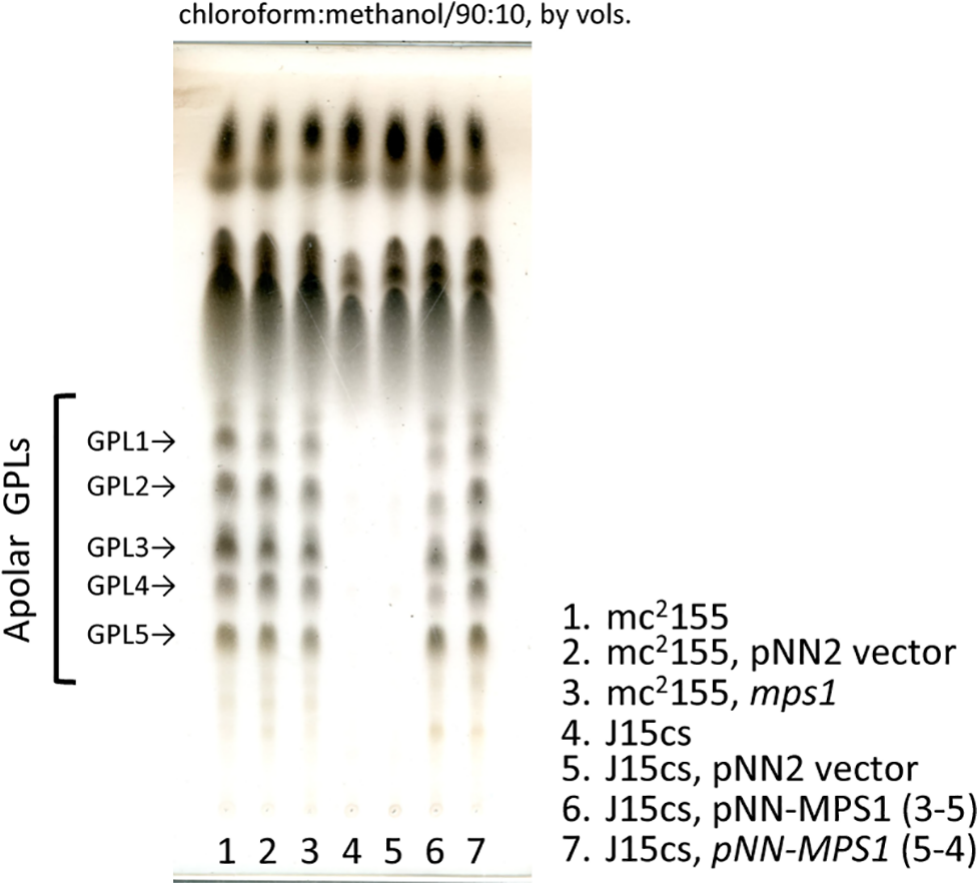
GPL phenotypes of *mps1* complement. Alkaline-stable lipids were developed with the solvent system chloroform-methanol (90:10, vol/vol) on TLC. The GPLs were restored in the *mps1*-complemented J15cs mutant. The TLC plate was sprayed with 20% sulfuric acid in ethanol and charred at 180°C for 3 min.

**Fig 4:**
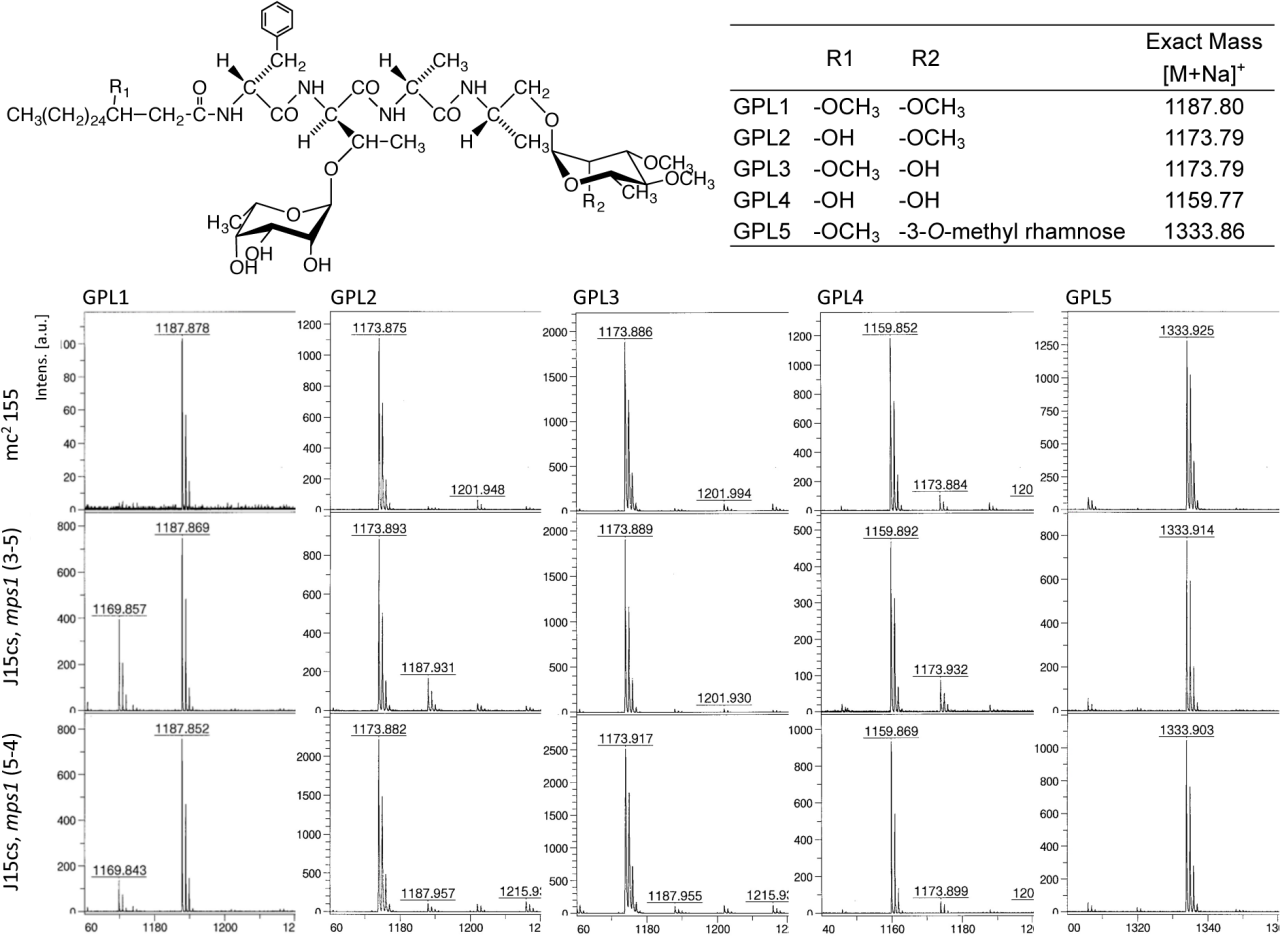
MALDI-TOF/MS spectra of each GPL. The molecular weights of GPLs derived from the *mps1*-complemented J15cs mutant were the same as those of the mc^**2**^155 strain. The matrix was 2,5-dihydroxybenzoic acid in chloroform-methanol (1:1, vol/vol). It was analyzed in the Reflectron mode with an accelerating voltage operating in positive mode at 20 kV. The main molecule-related ions were detected as m/z, [M+Na]^**+**^. Intens., intensity; a.u., arbitrary units. The mass numbers were fixed to the chemical structures and exact mass numbers.

Next, the *mps1* of the mc^2^155 strain (mc^2^155-*mps1*) was inserted into a pNN2 vector (kanamycin-resistant, Fig 2), and the J15cs strain was transformed using this plasmid by electroporation. The promoter of *aphII* was employed to express *mps1*. Two mutants (3–5, 5–4) of the J15cs strain, which were complemented with the mc^2^155-*mps1*, were obtained. The phenotypes of GPLs were examined. The alkaline-stable lipids were analyzed by TLC and are shown in Fig 3. Although the parent strain and the pNN2-vector-inserted J15cs mutant lacked GPL spots, the five major GPL spots of the *mps1*-complemented J15cs mutants were restored with the same Rf (retention factor) values on TLC as those of the mc^2^155 strain. In addition to the comparative Rf value, the molecular weight of each spot was measured by MALDI-TOF/MS (Fig 4). The mass spectra of the *mps1*-complemented J15cs mutants showed the same masses as the GPL1-5 derived from the mc^2^155 strain previously reported by Miyamoto et al. [11]. As a result, it was clarified that mutation of the *mps1* gene directly caused the deletion of GPLs in the J15cs strain.

### Diversity of morphology by GPLs restoration

**Fig 5:**
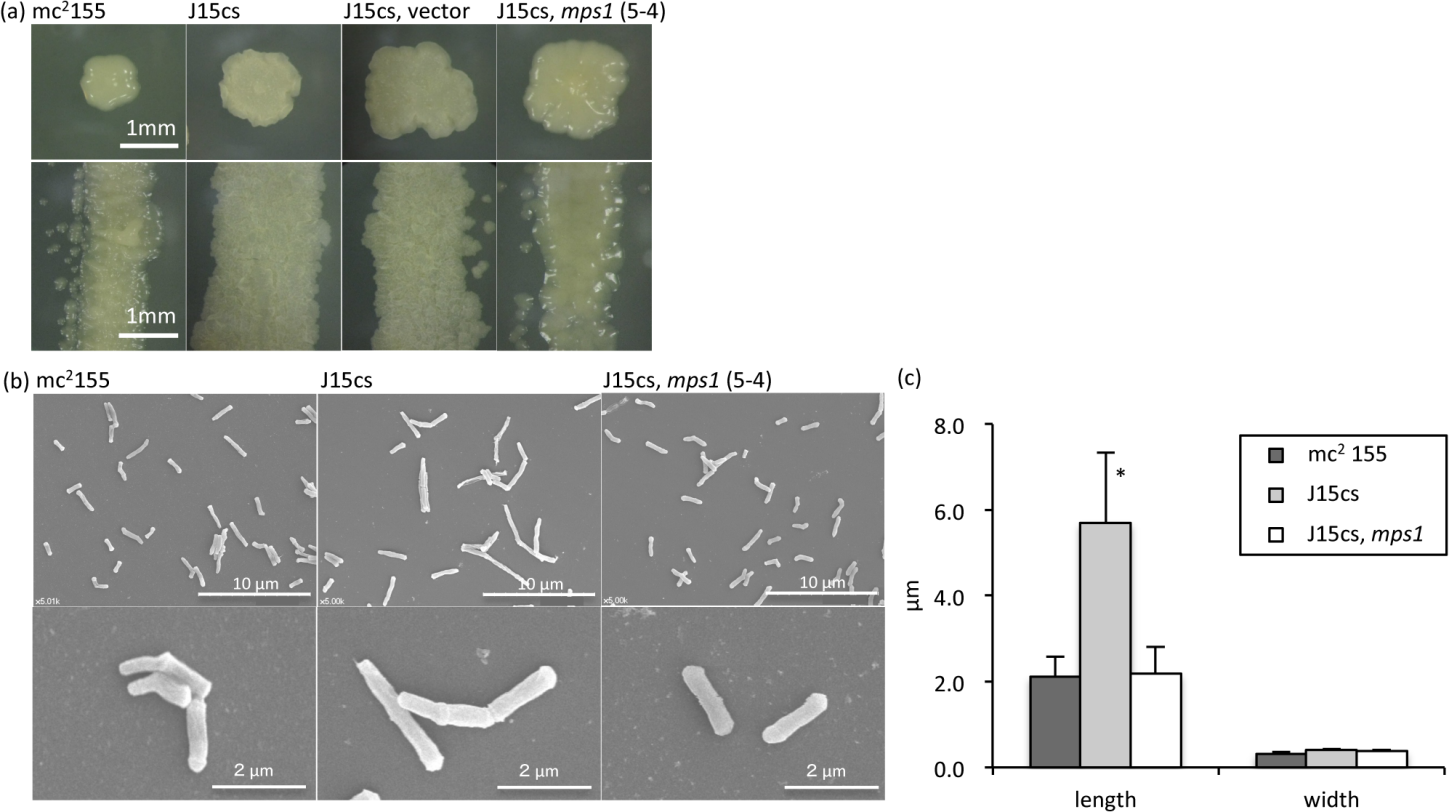
Change of morphology due to expression of GPLs. The colony morphology was observed by an optical microscope after culturing. The J15cs strain formed rough, dry, and irregular colonies. The mc^**2**^155 strain and the *mps1*-complemented J15cs mutant colonies were smoothly curved and wet (a). Scanning electron microscopy (b) and a comparison of bacterial cell length (c) are shown. The bacteria were grown on nutrient agar plates for 10 days at 37°C, collected, and filtered through a 5 μm membrane to remove aggregates. The average bacterial cell length was 2.11±0.46 μm, the mc^**2**^155 strain; 5.70±1.65, the J15cs strain; 2.18±0.62 μm, the *mps1*-complemented J15cs mutant. The data are means±standard deviations (SD) for 20 bacteria. The separate experiments were done in duplicate. *, *p<*0.001.

After being cultured on nutrient agar for 10 days at 37°C, the J15cs strain formed rough, dry, and irregular colonies. In contrast, the colonies of the mc^2^155 strain were smoothly curved and wet. Thus, the morphology of the colonies was strain-specific. The J15cs strain carrying the pNN2 vector showed the same morphology as the parent J15cs strain, and the *mps1*-complemented J15cs mutant formed smoothly curved and wet colonies like the mc^2^155 strain (Fig 5A). The hydrophobicity of the *mps1*-complemented J15cs mutant was reduced, compared to the parent J15cs strain. The SEM images showed that the bacterial length of the J15cs strain was longer than that of the mc^2^155 strain, and the *mps1*-complemented J15cs mutant was significantly shorter than the parent J15cs strain. There was no significant difference in their widths (Fig 5B and 5C). The restoration of GPLs in the *mps1*-complemented J15cs mutant may modify the cell wall construction. As a result, the colony morphology and bacterial size were changed to those of the mc^2^155 strain. It was concluded that the existence of GPLs affected bacterial size and morphology.

### Effect of GPLs on intracellular survival of host cells

**Fig 6:**
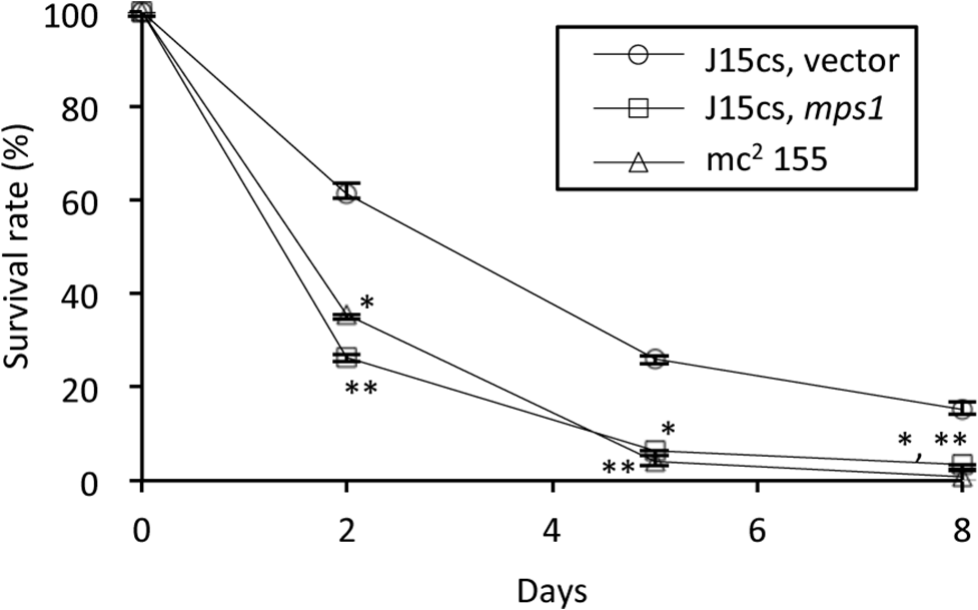
Survival of *mps1*-complemented J15cs mutant in J774.1 cells. The J774.1 cells were grown on 24-well flat-bottom tissue culture plates. A bacterial single-cell suspension of approximately 1×10^**7**^ CFU/well was inoculated into the J774.1 cells at an approximate multiplicity of infection 10. After cocultivation for 3 h, the infected J774.1 cells were washed, and the infected J774.1 cells were further incubated for 2 h with the medium plus 200 μg/ml gentamicin. The medium was replaced with fresh medium containing 2 μg/ml of gentamicin (this time point was day 0), and the infected cells cultured for 8 days. At various time intervals after inoculation, the adherent cells were treated with 1% Triton-X100/PBS, and were sonicated. The bacterial burden was evaluated by counting CFU. The separate experiments were done in triplicate, and statistical analysis was performed using Dunnett’s test. * and ** indicate a *p* value of <0.05, compared to the J15cs strain inserted with a pNN2 vector only respectively.

To determine the effect of GPLs on bacterial survival in host cells, J774.1 cells were infected with the *mps1*-complemented J15cs mutant. Surprisingly, this GPL-restored J15cs mutant did not survive as long as the parent J15cs strain. On day 2 after infection, the survival rate of the GPL-restored J15cs mutant was significantly lower than that of the parent strain, and the survival curve was almost the same as that of the mc^2^155 strain that expressed GPLs (Fig 6).

## Discussion

*M*. *smegmatis* is a widely analyzed mycobacterial infection model because of its nonpathogenicity and rapid growth. The J15cs strain survives in host cells longer than the mc^2^155 strain. In our previous reports, it was shown that the difference between these two strains was the presence or absence of an apolar GPL. GPLs are synthesized by some nontuberculous mycobacteria. Because of their location on the cell surface, their presence is considered to correlate with the pathogenicity of the bacteria by interfering with the host immune responses. We predicted that the GPLs play crucial roles in the intracellular survival of the J15cs strain. The GPL was composed of a tetrapeptide-linked acyl chain and a sugar. Recently, the biosynthesis pathway of GPL is well understood, and more than fifteen genes are involved [6,12]. In this study, we focused on the *mps1* and *mps2* genes, which belong to the non-ribosomal protein synthesis family (*nrp*), and are responsible for synthesis of the peptidyl core of the GPLs [13,14]. Comparison of the sequences shows that 18 bps encoding 6 amino acids were deleted in the *mps1* in the J15cs strain, and insertion of mc^2^155-*mps1* into the J15cs mutant restored expression of the GPLs. The MALDI-TOF/MS analysis showed that the detailed structure, modifying groups of acyl chains, the sugar of GPLs, and the GPLs of the *mps1*-complemened J15cs mutant were completely identical to those of the mc^2^155 strain. These results implied that the *mps1* mutation directly caused the deletion of GPLs in the J15cs strain. Because the deletion of 18 bps did not cause a frame-shift mutation, these deleted 6 amino acids in the Mps1 protein are considered to be important for the secondary, tertiary protein structures and active domain. Based on the phenotype of the *mps1*-complemented J15cs mutant, the deletion of GPLs affects two prominent features in the J15cs strain. One is bacterial size. The J15cs strain is longer than mc^2^155, and that of the *mps1*-complemented mutant was almost the same length as mc^2^155 rather than the parent J15cs strain. The other is the colony morphology. The J15cs strain was the rough type, and the complemented mutant shifted to the smooth type. These phenomena were due to the presence of GPLs.

Next, we were interested in whether these phenotypes correlated with bacterial survival in host cells or not. Surprisingly, the *mps1*-complemented J15cs mutant did not survive as long as the parent J15cs strain in host cells. This result showed that the survival of the J15cs strain is correlated with the lack of GPLs. Toll-like receptor (TLR) 2 recognizes GPLs [2,15,16,17]. The GPL-recognition via TLR2 does not work when the J15cs strain infects to host cell. As a result, the J15cs strain evades one of the major host-pathogen recognition systems and may survive longer than the other strain. In addition, the colony morphology and bacterial size may affect host-bacteria interaction. *Mycobacterium abscessus* is a rapidly growing mycobacteria closely related to *M*. *smegmatis*. This species is recognized as a pulmonary pathogen in humans. Recently, Bernut et al. reported that the rough variant of *M*. *abscessus*, which is devoid of GPLs, causes more severe clinical disease than the smooth variant. The mechanism of the increased virulence of the *M*. *abscessus* rough variant was explained to be the massive production of serpentine cords, which are absent in smooth variant infection. In apoptotic macrophages, the rough variant of *M*. *abscessus* initiated cord formation and grew so large that the macrophages and neutrophils could not phagocytize them [18,19]. In addition, Davidson et al. reported that GPLs mask underlying bioactive cell wall lipids, and the GPL-lacking *M*. *abscessus* rough variant is immunostimulatory and invasive in macrophage infection [15]. Roux et al. reported that the *M*. *abscessus* rough variant is associated with synthesis/exposure of the lipoprotein at the cell surface [20]. Etienne et al. reported that the GPL-deficient mc^2^155 mutant strain exhibited rough colony morphology, and indicated that GPLs are involved in the cell wall permeability barrier of *M*. *smegmatis* [21,22]. These previous reports show the possibility of the GPL-host relationship, and we summarize the reasons for the survival of the J15cs strain. By the deletion of GPLs, the J15cs strain lost prominent features that are correlated with host-bacterial interaction and host responses, in addition to the direct host immune response to GPLs via TLR2.

As for the GPLs, the major nontuberculous pathogens *M*. *avium* and *M*. *intracellulare* produce a serotype-specific polar GPL that plays a crucial role in the host immune responses. According to our previous report, the acetyl or methyl modifications of oligosaccharides are necessary for host cells to recognize polar GPLs via TLR2. The activities of the polar GPLs are greatly decreased by the deletion of acetyl groups from oligosaccharides [16]. The major epitopes for the host immune system may be oligosaccharides in the polar GPLs. The production of an anti-GPL IgA antibody is increased in patients and useful in the serodiagnosis of nontuberculosis infection using the GPL core antigen [23,24]. In the nontuberculous mycobacteria infection, the GPLs were one of the key components on the cell wall related to their virulence and pathogenicity. We should pay attention to the functions of two types of GPLs, the apolar types in *M*. *abscessus* and the polar types in the *M*. *avium-intracellulare* complex. On the other hand, GPLs are proposed to confer the capacity for sliding motility, biofilm formation, and dormancy (to be published elsewhere) [25,26].

In this study, direct evidence was shown that the *mps1* mutation causes the deletion of GPLs in the J15cs strain, and affects bacterial size, morphology, and survival in host cells.
